# Ambient Particulate Matter Induces Vascular Smooth Muscle Cell Phenotypic Changes via NOX1/ROS/NF-κB Dependent and Independent Pathways: Protective Effects of Polyphenols

**DOI:** 10.3390/antiox10050782

**Published:** 2021-05-14

**Authors:** Chia-Chi Ho, Yu-Cheng Chen, Ming-Hsien Tsai, Hui-Ti Tsai, Chen-Yi Weng, Shaw-Fang Yet, Pinpin Lin

**Affiliations:** 1 National Institute of Environmental Health Sciences, National Health Research Institutes, Zhunan 53053, Taiwan; 020604@nhri.org.tw (C.-C.H.); yucheng@nhri.org.tw (Y.-C.C.); mhtsai@nhri.org.tw (M.-H.T.); ava-jacky@nhri.org.tw (H.-T.T.); 030202@nhri.org.tw (C.-Y.W.); 2 Institute of Cellular and System Medicine, National Health Research Institutes, Zhunan 53053, Taiwan

**Keywords:** ambient particulate matter, vascular phenotypic changes, migration, inflammation, oxidative stress, NADPH oxidase 1, NF-κB

## Abstract

Epidemiological studies have demonstrated an association between ambient particulate matter (PM) exposure and vascular diseases. Here, we observed that treatment with ambient PM increased cell migration ability in vascular smooth muscle cells (VSMCs) and pulmonary arterial SMCs (PASMCs). These results suggest that VSMCs and PASMCs transitioned from a differentiated to a synthetic phenotype after PM exposure. Furthermore, treatment with PM increased intracellular reactive oxygen species (ROS), activated the NF-κB signaling pathway, and increased the expression of proinflammatory cytokines in VSMCs. Using specific inhibitors, we demonstrated that PM increased the migration ability of VSMCs via the nicotinamide–adenine dinucleotide phosphate (NADPH) oxidase 1 (NOX1)/ROS-dependent NF-κB signaling pathway, which also partially involved in the induction of proinflammatory cytokines. Finally, we investigated whether nature polyphenolic compounds prevent PM-induced migration and proinflammatory cytokines secretion in VSMCs. Curcumin, resveratrol, and gallic acid prevented PM_2.5_-induced migration via the ROS-dependent NF-κB signaling pathway. However, honokiol did not prevent PM_2.5_-induced migration or activation of the ROS-dependent NF-κB signaling pathway. On the other hand, all polyphenols prevented PM_2.5_-induced cytokines secretion. These data indicated that polyphenols prevented PM-induced migration and cytokine secretion via blocking the ROS-dependent NF-κB signaling pathway in VSMCs. However, other mechanisms may also contribute to PM-induced cytokine secretion.

## 1. Introduction

Ambient particulate matter (PM) is a widespread air pollutant. Numerous epidemiological studies have demonstrated that exposure to ambient PM with a diameter lower than 2.5 (PM_2.5_) or 10 μm (PM_10_) is associated with morbidity, mortality, and hospitalization due to pulmonary and cardiovascular diseases [1,2,3]. However, the critical pathophysiological mechanisms of PM-induced vascular diseases have not been fully elucidated.

The human arteries are composed of tunica intima, tunica media, and tunica adventitia. Carotid intima–media thickness (IMT) has been widely used as an indicator of early atherosclerosis [4]. Epidemiological studies in the United States, Europe, and Taiwan have demonstrated an association between long-term exposure to PM_10_ or PM_2.5_ and increased carotid IMT [5,6,7,8]. In addition, some epidemiological studies have demonstrated an association between PM_2.5_ exposure and elevated blood pressure [9,10] and pulmonary arterial pressure [11,12]. Animal studies have reported that exposure to PM increased blood pressure [13,14]. Therefore, we hypothesized that vascular cells are targets of PM.

Vascular smooth muscle cells (VSMCs) are located in the tunica media of an artery. Under normal physiological conditions, VSMCs within the arterial wall exhibit a highly differentiated (contractile) phenotype. However, in response to various stimuli, differentiated VSMCs can switch to a “dedifferentiated” (also termed “synthetic”) phenotype, exhibiting increased proliferative and migratory ability, downregulation of VSMC marker expressions, and increased proinflammatory proteins secretion [15]. For example, studies have reported that cigarette smoke particles changed the VSM phenotype and promoted the proliferation of VSMCs and airway SMCs [16,17]. We reported that exposure to ambient PM_2.5_ increases the proliferation of VSMCs and cytokines secretion in VSMCs [18]. Thus, we propose that exposure to ambient PM may cause VSMC phenotypic changes. However, the possible mechanism remains unclear.

Vascular inflammation and oxidative stress have been suggested to play roles in the pathogenesis of cardiovascular diseases such as hypertension and atherosclerosis [19,20]. We reported that exposure to ambient PM_2.5-10_ caused pulmonary and systemic inflammation and increased proinflammatory cytokines secretion in mice [13]. Furthermore, exposure to ambient PM_2.5_ not only caused pulmonary inflammation but also increased urinary 8-hydroxydeoxyguanosine levels in mice [21]. In cultivated VSMCs, we demonstrated that exposure to PM_2.5_ or PM_2.5-10_ increased reactive oxygen species (ROS) levels and proinflammatory cytokine secretion [18,21]. Therefore, investigating the roles of inflammation and oxidative stress in ambient PM-induced vascular phenotypic changes is worthwhile. 

Polyphenols are a kind of herbal compound that offer various functions, many of which possess powerful antioxidative, anti-inflammatory, and anticancer activities [22]. Polyphenols are beneficial compounds in many plants and are divided into flavonoids, phenolic acids, polyphenol amides and other polyphenols. For example, the administration of curcumin, an antioxidative and anti-inflammatory polyphenolic compound from the spice turmeric, was significantly associated with improvement in triglycerides, high-density lipoprotein cholesterol, and diastolic blood pressure levels in an epidemiological study [23]. Resveratrol, a polyphenolic compound in red wine with antioxidant properties, reduced weight, triglyceride levels, and systolic blood pressure (SBP) in rats [24]. Gallic acid (GA), a phenolic acid, has anti-inflammatory, antiangiogenic, antioxidative, and anticancer activities [25]. Oral administration of GA attenuated ROS and reduced aortic wall thickness and SBP in spontaneously hypertensive rats [26]. If oxidative stress and inflammation are involved in the mechanism mediating ambient PM exposure related cardiovascular diseases, antioxidative and anti-inflammatory herbal compounds may be useful for preventing PM-induced vascular toxicity. 

The present study elucidated the mechanisms of ambient PM-induced vascular phenotypic changes. We observed that ambient PM_2.5_ and PM_2.5-10_ caused VSMC phenotypic changes, namely increased migratory ability and proinflammatory cytokines secretion. On the basis of our research, we studied the roles of ROS-related mechanisms in ambient PM-induced VSMC phenotypic changes. Finally, we evaluated the preventive effects of herbal compounds in PM_2.5_-induced migration and proinflammatory cytokine secretion in VSMCs. Our findings suggest that antioxidation is a mechanism-based approach to prevent PM-induced vascular phenotypic changes and may facilitate the development of strategies for preventing ambient PM-induced vascular diseases.

## 2. Materials and Methods

### 2.1. Materials

PS-1145 (IKK inhibitor) and GKT13781 (NOX inhibitor) were purchased from Cayman Chemical Company (Ann Arbor, MI, USA). ML171 (NOX1 inhibitor) was purchased from Tocris Bioscience (Minneapolis, MN, USA). *N*-acetyl-l-cysteine (NAC) was purchased from Sigma (St Louis, MO, USA).

### 2.2. PM Sample Collection

PM samples were collected in November 2016 in Kaohsiung City (KH), Taiwan, through two-stage (PM_2.5_ and PM_2.5-10_) high-volume impaction (ChemVol model 2400, BGI, Inc., Waltham, MA, USA) at 900 L/min. PM_2.5_ and PM_2.5–10_ samples were collected on fiberglass filters coated with polyvinylidene difluoride (Pallflex Fiberfilm T60A20; Pall Corporation, New York, NY, USA) and polyurethane foam (PUF) substrates (McMaster-Carr, Atlanta, GA, USA), respectively. Characteristics and chemical components of PM_2.5_ and PM_2.5-10_ samples collected in 2016 were described previously [18]. In addition, we collected individual PM_2.5_ samples from January to March 2018 in KH through high-volume impaction by using a Digitel DHA-80 aerosol sampler (Digitel, Hegnau, Switzerland) at 500 L/min. PM_2.5_ samples were collected on fiberglass filters coated with polyvinylidene difluoride (Pallflex Fiberfilm TX40HI20; Pall Corporation, New York, NY, USA). Characteristics and chemical components of PM_2.5_ samples collected in 2018 were described [21]. All samples were stored at −20 °C after collection. Prior to and following each collection, the fiberglass filters and PUF substrates were weighed using standard operating procedures in an environmentally controlled room (23 ± 1 °C with a relative humidity 40% ± 5%) on an analytical balance (AG204 dual-range, Mettler Toledo, Columbus, OH, USA) to determine the amount of collected PM. PM_2.5_ and PM_2.5-10_ samples collected simultaneously in 2016 were used for determining the contributing mechanism, and PM_2.5_ samples collected in 2018 were used to study the preventive effects of the herbal compounds.

### 2.3. PM Extracts

Fiberglass filters used for collecting PM_2.5_ were wetted with 70% ethanol in a glass measuring beaker and subsequently sonicated for 30 min at room temperature. The PUF substrates used for collecting PM_2.5-10_ were wetted using double-distilled water (d_2_H_2_O) in a glass measuring beaker and subsequently sonicated for 1 h at room temperature. PM_2.5_ and PM_2.5-10_ were extracted as previously described [18].

### 2.4. Animal Experiments

Six-week-old male mice (C57BL/6J) were purchased from the National Laboratory Animal Center (Taipei, Taiwan) and housed at the National Health Research Institutes (NHRI). All animal treatments and experimental protocols (NHRI-IACUC-106003-A) were reviewed and approved by the Institutional Animal Care and Use Committee of the NHRI. All mice were housed under a 12-h light–dark cycle at 23 °C ± 1 °C, with a relative humidity of 39–43%. Water and food were provided ad libitum. Eight-week-old mice were divided into groups exposed to d_2_H_2_O as control or 25 μg of PM_2.5-10_ for 2, 4, and 8 weeks and groups exposed to 25 μg of PM_2.5_ for 8 weeks. Ten mice (*n* = 10) from each group were randomly selected for experimentation. Animals were exposed to PM twice per week by oropharyngeal aspiration. 

### 2.5. Oropharyngeal Aspiration

Mice were anesthetized through isoflurane inhalation. While under anesthesia, each mouse was secured on its back on an inclined plane with its head elevated. The mouth was secured in an open position with a rubber band, and the tongue was held to one side by using forceps for visualization of the epiglottis. A syringe fitted with a blunt, polished needle (19 gauge, 3 inches long, angled at 45°) was inserted into the mouth until it reached the larynx. The sample was then rapidly expelled. The mice were given 30 μL of distilled water or 25 μg of PM_2.5_ or PM_2.5-10_ per 30 μL of water. 

### 2.6. Preparation of Bronchoalveolar Lavage Fluid

The mice were sacrificed through inhalation of isoflurane. The lungs were lavaged with 1 mL of saline, and the recovered amount of lavagate was recorded and stored. The total cell number and cell type distribution in the bronchoalveolar lavage fluid (BALF) were determined as described [27].

### 2.7. Total Protein Concentration and Lactate Dehydrogenase Activity in BALF

Total protein concentrations in the BALF supernatant were determined using the Bradford assay (Bio-Rad, Hercules, CA, USA) with bovine serum albumin as the standard. The lactate dehydrogenase (LDH) activity was spectrophotometrically measured using the CytoTox96 Non-Radioactive Cytotoxicity Assay (Promega Corporation, Madison, WI, USA) at 490 nm in the presence of lactate.

### 2.8. Histological Analysis 

We isolated the left lobe and right inferior lobe of the lung and horizontally cross sectioned the middle part of the lobes, which contained secondary bronchi, bronchioles, alveolar ducts and sac. Hematoxylin and eosin (H&E) stain was performed for histopathological examinations according to a previously described protocol [28]. Immunohistochemistry was based on a previously described protocol [29]. In brief, the antibody was for smooth muscle α-actin (SMA) (Sigma-Aldrich A5228, St. Louis, MO, USA). Sections were stained with elastic stain kit (Sigma-Aldrich, St. Louis, MO, USA) for elastin fiber or the Trichrome Stain (Masson) Kit (Sigma-Aldrich, St. Louis, MO, USA) for collagen fiber according to the manufacturer’s instructions.

### 2.9. Cell Culture

Primary mouse VSMCs (MVSMCs) were isolated from mouse aortas and cultured in Dulbecco’s Modified Eagle Medium (GIBCO^TM^, Carlsbad, CA, USA) with L-glutamine, sodium bicarbonate, and fetal bovine serum (FBS) as described [30]. Human pulmonary artery SMCs (HPASMCs) were purchased from Lonza (Walkersville, MD, USA) (Cat# CC2581) and cultured in SMC medium (Lonza, Walkersville, MD, USA; cat# CC3182). Mouse pulmonary artery SMCs (MPASMCs) were purchased from Cell Biologics Inc. (Chicago, IL, USA; cat# C57-6083) and cultured in SMC medium (Cell Biologics Inc., Chicago, IL, USA; cat# M2268). Both cells at passages 5–8 were used in the following experimental assays and were incubated at 37 °C in a humidified mixture of 5% CO_2_ and 95% air.

### 2.10. Quantitative Real-Time Reverse Transcription–Polymerase Chain Reaction Assays

Total RNA was prepared using RNAzol reagent (Life Technologies, Rockville, MD, USA). The cDNA was synthesized using a High-Capacity cDNA Archive Kit (P/N4322171, Applied Biosystems, Foster City, CA, USA). The quantitative polymerase chain reaction (qPCR) assays were conducted using a TaqMan Universal PCR Master Mix (Applied Biosystems, Foster City, CA, USA) and an ABI StepOnePlus^TM^ real-time PCR system (Perkin–Elmer Applied Biosystems, Foster City, CA, USA). The relative mRNA levels of the target gene are presented as previously described [31].

### 2.11. Lamellipodia Formation

The mVSMCs (2 × 10^4^ cells) were seeded on cover slides and subsequently treated with vehicle (double distilled water, d_2_H_2_O) or 25 μg/mL KH PM_2.5_ or PM_2.5-10_ for 48 h in 0.5% FBS medium. The cells were fixed with 4% paraformaldehyde at room temperature for 10 min, washed with phosphate buffer saline (PBS), and then stained with Alexa Fluor 546 phalloidin (Invitrogen, Emeryville, CA, USA) for F-actin at room temperature for 60 min. Cells were observed under Leica DMRXA fluorescence microscopy.

### 2.12. Migration Assay

Cells were treated with vehicle or 25 μg/mL KH PM_2.5_ or PM_2.5-10_ with and without inhibitors in 0.5% FBS medium for 48 h. Cells were placed in the upper chamber of 24-well Transwell cell culture plates (Millipore, Burlington, MA, USA; 8-μm pore size). The bottom chambers were filled with media containing platelet-derived growth factor (PDGF)-BB (Peprotech, Rocky Hill, NJ, USA; 10 ng/mL) as a chemoattractant. After incubation for 4 h in MVSMC and MPASMC or 16 h in HPASMC, the upper layer of cells was scraped off, and the membrane was fixed and stained with crystal violet (Sigma-Aldrich, St. Louis, MO, USA). Cells that migrated to the underside of the membrane were visualized using a microscope. Image analyses were performed using MetaMorph 7.8.11.0 software.

### 2.13. Intracellular ROS Assay

VSMCs were seeded in 96-well plates and treated with vehicle or 12.5 μg/mL KH PM_2.5_ or PM_2.5-10_ with and without inhibitors in 0.5% FBS medium. The intracellular ROS level was determined by measuring the conversion of fluorescent dichlorofluorescein (DCF) from dichloro–dihydro–fluorescein diacetate (H_2_DCFDA) through ROS-mediated oxidation; 100 μM of H_2_DCFDAwas added to the cells 60 min before treatment with PM_2.5_. DCF fluorescence was measured using the SpectraMax i3x Multi-Mode Microplate Reader (Molecular Devices, Sunnyvale, CA, USA), with excitation and emission at 485 nm and 525 nm, respectively.

### 2.14. Mitochondria ROS Assay

VSMCs (2 × 10^4^ cells) were seeded on cover slides and subsequently treated with vehicle or 12.5 μg/mL KH PM_2.5_ or PM_2.5-10_ for 24 h in 0.5% FBS medium. The cells were fixed with 4% paraformaldehyde at room temperature for 10 min, washed with PBS, and then stained with the MitoSOX Red reagent (Invitrogen, Emeryville, CA, USA) for 10 min. Cells were observed under Leica DMRXA fluorescence microscopy.

### 2.15. Enzyme-Linked Immunosorbent Assay

The chemokine (C–X–C motif) ligand 1 (CXCL1) and interleukin-6 (IL-6) concentration in the medium were measured using enzyme-linked immunosorbent assay kits for mice (R&D Systems, Inc., Minneapolis, MN, USA) in accordance with the manufacturer’s instructions. 

### 2.16. NF-κB Reporter Gene Assay

For the luciferase assays, VSMCs were transfected with the pNF-κB-Luc and pCMV-β-gal using Lipofectamine 2000 (Invitrogen, Emeryville, CA, USA) according to the manufacturer’s protocol. The transcriptional activity was determined using the Luciferase Assay System (Promega, Madison, WI, USA) and a luminometer (Berthold Analytical Instruments, Nashua, NH, USA). 

### 2.17. NOX4 RNA Interference Knockdown

VSMCs were seeded in 96-well plates or a 6-cm dish and treated with vehicle or 12.5 μg/mL KH PM_2.5_ or PM_2.5-10_ for 48 h in 0.5% FBS medium. The NOX4 target sequence used was AAAAGCAAGACTCTACACATC. The negative control sequence used was AATTCTCCGAACGTGTCACGT. VSMCs were transfected with the siRNA duplexes (5 pmole in 96-well or 200 pmole in a 6-cm dish) by using Lipofectamine 2000 (Invitrogen, Emeryville, CA, USA) following the manufacturer’s instructions.

### 2.18. Statistical Analysis

Statistical analyses were performed using SPSS 15.0 software. The treatment and control groups were compared using one-way analysis of variance, followed by Tukey’s range test in SPSS Statistics (significance: *p* < 0.05). Groups of 2, 4 and 8 weeks’ exposure in the animal experiment were compared using two-way analysis of variance followed by Tukey’s range test in SPSS Statistics (significance: *p* < 0.05).

## 3. Results

### 3.1. Exposure to Ambient PM Caused Different Time-Dependent Pulmonary Inflammation and Vascular Injury Changes in Mice

**Figure 1:**
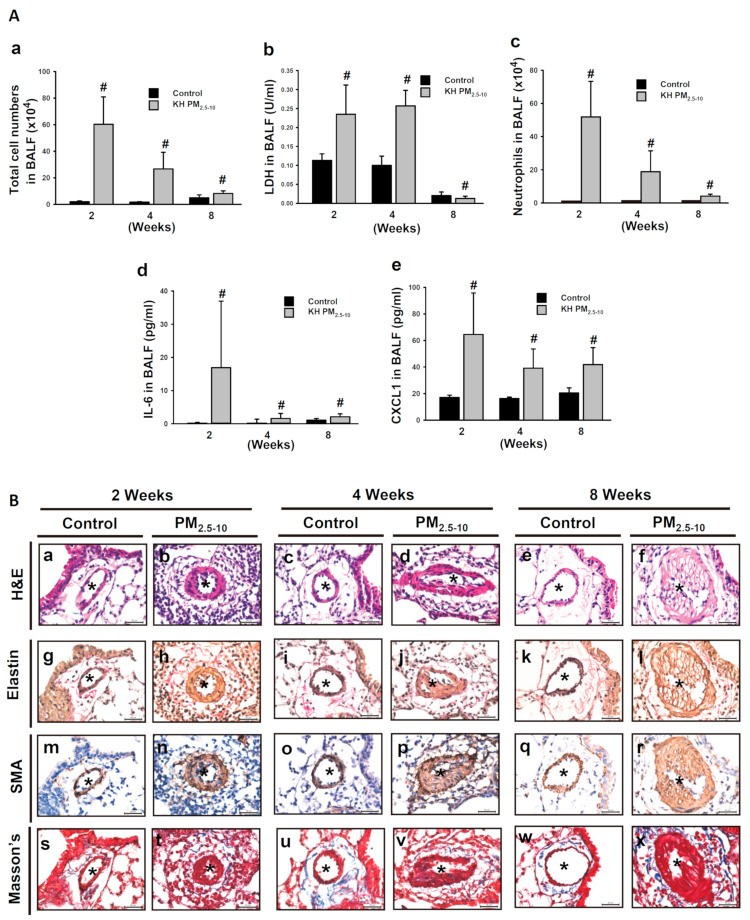
PM_2.5-10_ induced pulmonary inflammation and vascular remodeling at 2, 4, and 8 weeks in mouse lungs. (**A**) Each mouse was aspirated with 25 μg of PM_2.5-10_ twice per week for 2, 4, and 8 weeks. (**a**) Total cell numbers, (**b**) LDH activity, (**c**) neutrophil numbers, (**d**) IL-6 protein, and (**e**) CXCL1 protein in BALF were determined. # *p* < 0.05, compared with respective control-treated mice. (**B**) Each mouse was aspirated with 25 μg of PM_2.5-10_ twice per week for 2, 4, and 8 weeks. H&E; Verhoeff’s staining; SMA staining; Masson’s Trichome Staining. Star indicates small arteries. Scale bar, 30 μm.

We have demonstrated that oropharyngeal aspiration of ambient PM_2.5_ and PM_2.5-10_ for 8 weeks induced pulmonary inflammation and resulted in medial thickening, which then progressed to intimal hyperplasia in pulmonary small arteries [13,18]. The time-course of PM-induced pulmonary inflammation and vascular alteration was further investigated in the present study. Oropharyngeal aspiration of 25 μg PM_2.5-10_ twice per week for 2 weeks induced acute lung inflammation with immune cells infiltration in mice, which persisted after repeated exposure for 4 and 8 weeks (data not shown). However, the increases in total cell number, LDH activity, neutrophil numbers, and CXCL1 and IL-6 levels in the BALF were statistically more drastic at week 2 than week 4 and 8 (Figure 1A, a–e). These results indicate that PM_2.5-10_-induced pulmonary inflammation declined over time. 

However, vascular alteration progressed and worsened after 2, 4, and 8 weeks following oropharyngeal aspiration of PM_2.5-10_. After H&E (Figure 1B, a–b) and elastin staining (Figure 1B, g–h), we observed medial thickening of the small arteries at 2 weeks. Exposure for 4 weeks further enhanced medial thickening (Figure 1B, c–d). At 8 weeks, we not only observed medial thickening, but also intimal hyperplasia (Figure 1B, e–f). The disappearance of the elastin layer in the pulmonary arterioles became more pronounced as exposure time increased (Figure 1B, g–h). Staining with smooth muscle marker α-SMA confirmed an increase in VSMCs in the intima and media of remodeled vessels (Figure 1B, m–r). Masson’s trichrome stain demonstrated an increase in collagen fiber deposition in the perivascular areas (Figure 1B, s–x). The increase in VSMCs and collagen fiber depositions in remodeled vessels became more obvious as exposure time increased. Similar vascular alterations and pulmonary inflammation [18] were observed in mice treated with PM_2.5_ for 8 weeks (Figure S1). Taken together, these results reveal that PM-induced vascular alterations progressed over time. Thus, vascular alteration was a major adverse effect following long-term exposure to PM.

### 3.2. Ambient PM Induced Cell Migration in PASMCs and VSMCs

**Figure 2:**
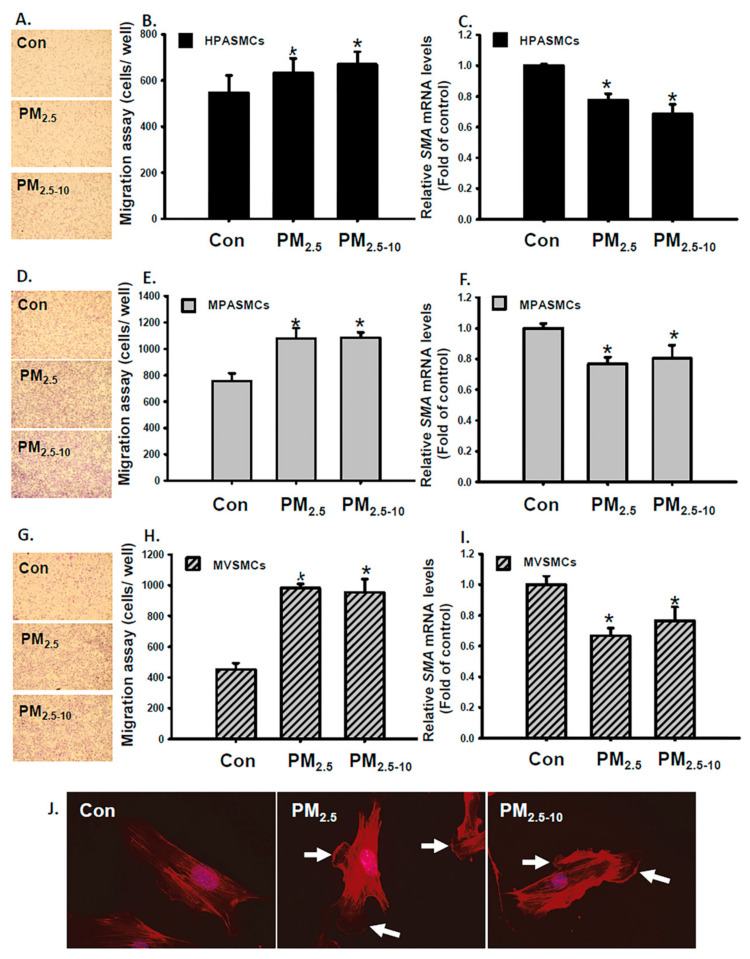
PM_2.5_ and PM_2.5-10_ induced cell migration in HPASMCs, MPASMCs, and MVSMCs. HPASMCs were treated with d_2_H_2_O or 25 μg/mL PM_2.5_ or PM_2.5-10_ for 48 h. Migration abilities of HPASMCs, MPASMCs, and MVSMCs were measured, quantified and shown as (**A**,**B**,**D**,**E**,**G**,**H**). The relative levels of SMA mRNA for HPASMCs, MPASMCs, and MVSMCs were determined using a real-time PCR assay, and shown as (**C**,**F**,**I**). (**J**) MVSMCs were stained with Alexa Fluor 546-Phalloidin.(red) to identify lamellipodia (white arrows). The results are presented as the mean ± SD for three or four independent experiments. * *p* < 0.05, compared with d_2_H_2_O-treated cells. Con: d_2_H_2_O treatment as control.

Given that the migration of medial VSMCs of the arteries contributes to intimal lesion formation, we surmised that PM_2.5-10_ and PM_2.5_ may affect VSMC functions such as migration. Therefore, we further investigated the effects of PM_2.5-10_ and PM_2.5_ on the functions of VSMCs and PASMCs in vitro. Treatment with PM_2.5-10_ and PM_2.5_ increased cell viability, enhanced migratory ability and reduced VSMC marker (SMA) expression were observed in HPASMCs, MPASMCs and MVSMCs (Figure 2, Figures S2 and S3). Furthermore, PM_2.5-10_ and PM_2.5_ increased membrane ruffles and lamellipodia formation in MVSMCs (Figure 2J). VSMC migration is a characteristic index of synthetic phenotype. These results support our in vivo data, suggesting that PM_2.5-10_ and PM_2.5_ caused VSMC phenotypic changes through phenotypic modulation into a synthetic phenotype.

### 3.3. Ambient PM Increased ROS Levels Through the NOX1 Dependent Pathway in MVSMCs

**Figure 3:**
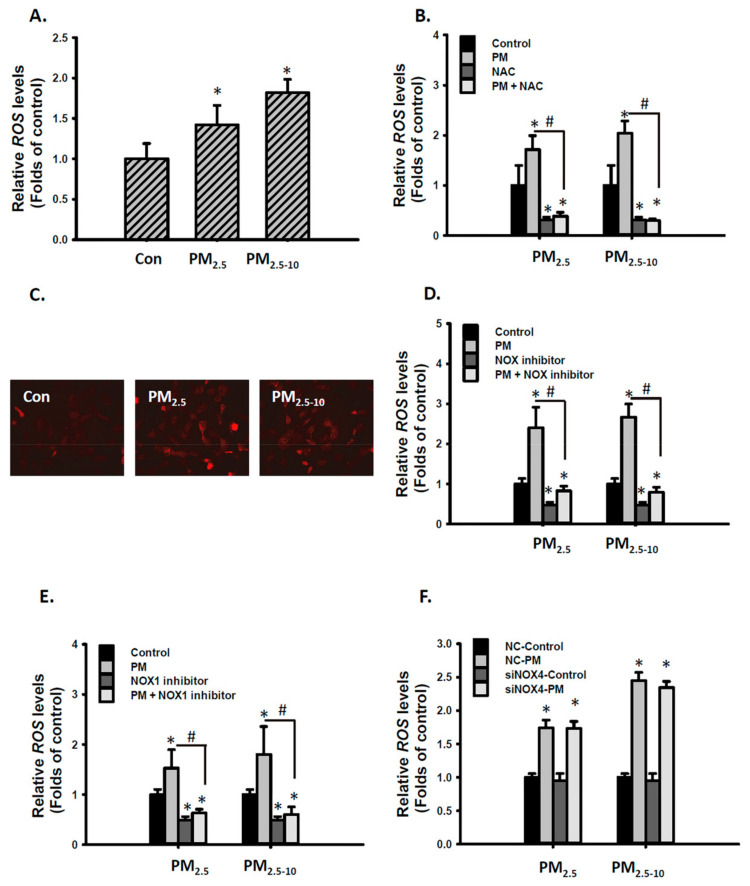
PM_2.5_ and PM_2.5-10_ increased ROS levels through the NOX1 dependent pathway in MVSMCs. (**A**) MVSMCs were treated with d_2_H_2_O or 12.5 μg/mL PM_2.5_ or PM_2.5-10_ for 2.5 h. (**B**) MVSMCs were treated with d_2_H_2_O or 12.5 μg/mL PM_2.5_ or PM_2.5-10_ with and without 10 μM NAC for 2.5 h. (**C**) MVSMCs were treated with d_2_H_2_O or 12.5 μg/mL PM_2.5_ or PM_2.5-10_ for 24 h, and mitochondrial ROS were detected with MitoSOX. (**D**) MVSMCs were treated with d_2_H_2_O or 12.5 μg/mL PM_2.5_ or PM_2.5-10_ with and without 10 μM of NOX inhibitor for 2.5 h. (**E**) MVSMCs were treated with d_2_H_2_O or 12.5 μg/mL PM_2.5_ or PM_2.5-10_ with and without 10 μM of NOX1 inhibitor for 2.5 h. (**F**) NC-MVSMCs and siNOX4-MVSMCs were treated with d_2_H_2_O or 12.5 μg/mL PM_2.5_ or PM_2.5-10_ for 2.5 h. Intracellular ROS levels were quantified using the cellular H_2_DCFDA assay. The results are presented as the mean ± SD for three or four independent experiments. * *p* < 0.05, compared with vehicle-treated cells. # *p* < 0.05, compared with inhibitor-treated cells. Con: d_2_H_2_O treatment as control; NC: negative control for siRNA.

ROS production is associated with vascular remodeling, including VSMC proliferation, hypertrophy, and migration [32]. ROS generated from air pollution exposure is suggested to be a crucial regulator of PM-induced cardiovascular diseases [33]. Our previous study has shown that PM_2.5_ exposure increased urinary 8-hydroxy-2′-deoxyguanosine concentrations in mice [21]. We further investigated whether PM increases ROS levels in MVSMCs. PM_2.5-10_ and PM_2.5_ significantly increased ROS levels in MVSMCs (Figure 3A). NAC, a common ROS inhibitor, completely prevented PM_2.5-10_- and PM_2.5_-increased ROS levels (Figure 3B). Mitochondrial-derived ROS is a source of cytosolic ROS. However, mitochondrial ROS levels only slightly increased in PM-treated MVSMCs (Figure 3C). Furthermore, mito-TEMPO, a mitochondria-specific superoxide scavenger, failed to influence PM-induced ROS production (data not shown), suggesting that the mitochondria are not major sources of cytosolic ROS. NADPH oxidases (NOXs) catalyze the production of a superoxide-free radical by transferring one electron from NADPH to oxygen and are critical determinants of the redox state in vascular systems [34]. Cotreatment with the NOX inhibitor completely blocked PM_2.5-10_- and PM_2.5_-increased ROS (Figure 3D). NOX1 and NOX4 are both expressed in human and rodent VSMCs [35]. To understand whether PM-increased ROS levels are mediated by NOX1 or NOX4, we inhibited NOX1 activity or knocked down NOX4 expression in MVSMCs. Inhibiting NOX1 activity completely blocked PM_2.5-10_- and PM_2.5_-increased ROS levels (Figure 3E), but reducing NOX4 expression through siRNA knockdown did not (Figure 3F). These results reveal that PM-increased intracellular ROS levels are mediated by NOX1 in MVSMCs.

### 3.4. Ambient PM Induced Cell Migration through the NOX1-ROS Signaling Pathway

**Figure 4:**
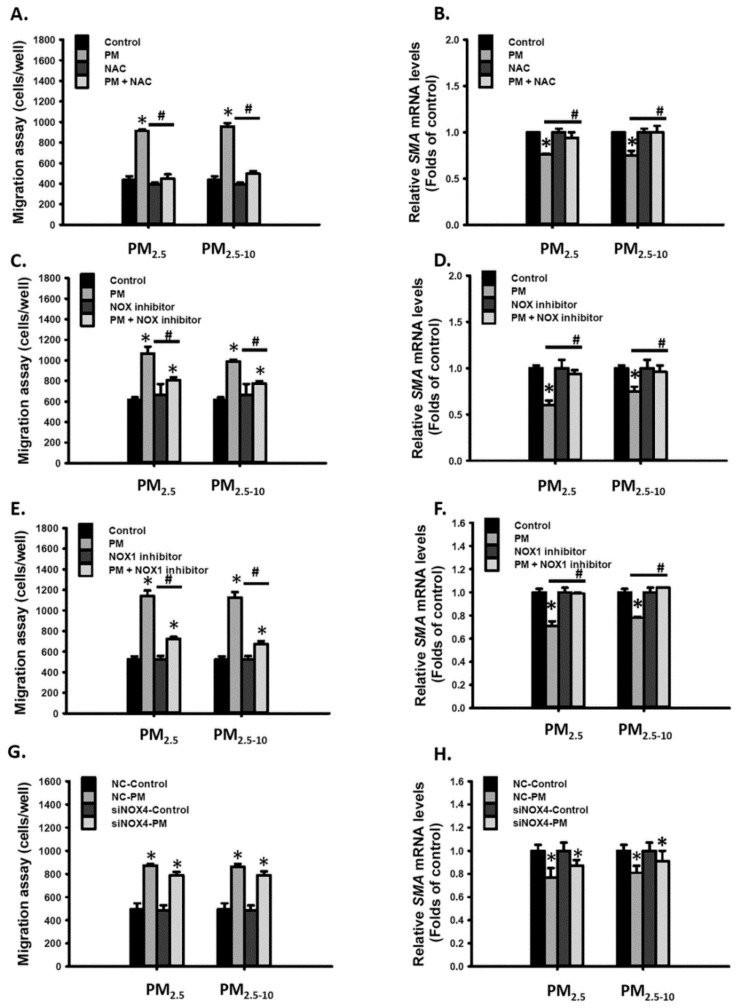
PM_2.5_ and PM_2.5-10_ induced cell migration via the NOX1-ROS signaling pathway in MVSMCs. MVSMCs were treated with d_2_H_2_O or 25 μg/mL PM_2.5_ or PM_2.5-10_ with and without 10 μM NAC for 48 h. (**A**) Cell migration ability and (**B**) relative SMA mRNA levels were determined. MVSMCs were treated with d_2_H_2_O or 25 μg/mL PM_2.5_ or PM_2.5-10_ with and without 10 μM NOX inhibitor for 48 h. (**C**) Cell migration ability and (**D**) relative SMA mRNA levels were determined. MVSMCs were treated with d_2_H_2_O or 25 μg/mL PM_2.5_ or PM_2.5-10_ with and without 10 μM NOX1 inhibitor for 48 h. (**E**) Cell migration ability and (**F**) relative SMA mRNA levels were determined. NC-MVSMCs and siNOX4- MVSMCs were treated with d_2_H_2_O or 12.5 μg/mL PM_2.5_ or PM_2.5-10_ for 48 h. (**G**) Cell migration ability and (**H**) relative SMA mRNA levels were determined. The results are presented as the mean ± SD for three or three independent experiments. * *p* < 0.05, compared with vehicle-treated cells. # *p* < 0.05, compared with inhibitor-treated cells. Con: vehicle treatment as control; NC: negative control for siRNA.

We further investigated whether ROS is involved in PM-induced cell migration. First, cotreatment with NAC completely blocked PM_2.5-10_- and PM_2.5_- enhanced migratory ability in MVSMCs (Figure 4A) and restored PM_2.5-10_- and PM_2.5_-reduced SMA mRNA levels (Figure 4B). Similarly, NOX inhibitor and NOX1 inhibitor completely blocked PM_2.5-10_- and PM_2.5_-enhanced migratory ability (Figure 4C,E) and restored PM_2.5-10_- and PM_2.5_-reduced SMA expression (Figure 4D,F). However, reducing NOX4 expression through siRNA knockdown did not inhibit PM_2.5-10_- and PM_2.5_-enhanced migratory ability or restore PM_2.5-10_- and PM_2.5_-reduced SMA expression (Figure 4G,H). According to these results, we concluded that PM_2.5-10_ and PM_2.5_ increased cell migration through the NOX1-ROS signaling pathway in MVSMCs.

### 3.5. The NOX1-ROS Signaling Pathway was Partially Involved in Ambient PM-Induced Inflammatory Cytokine Expression

**Figure 5:**
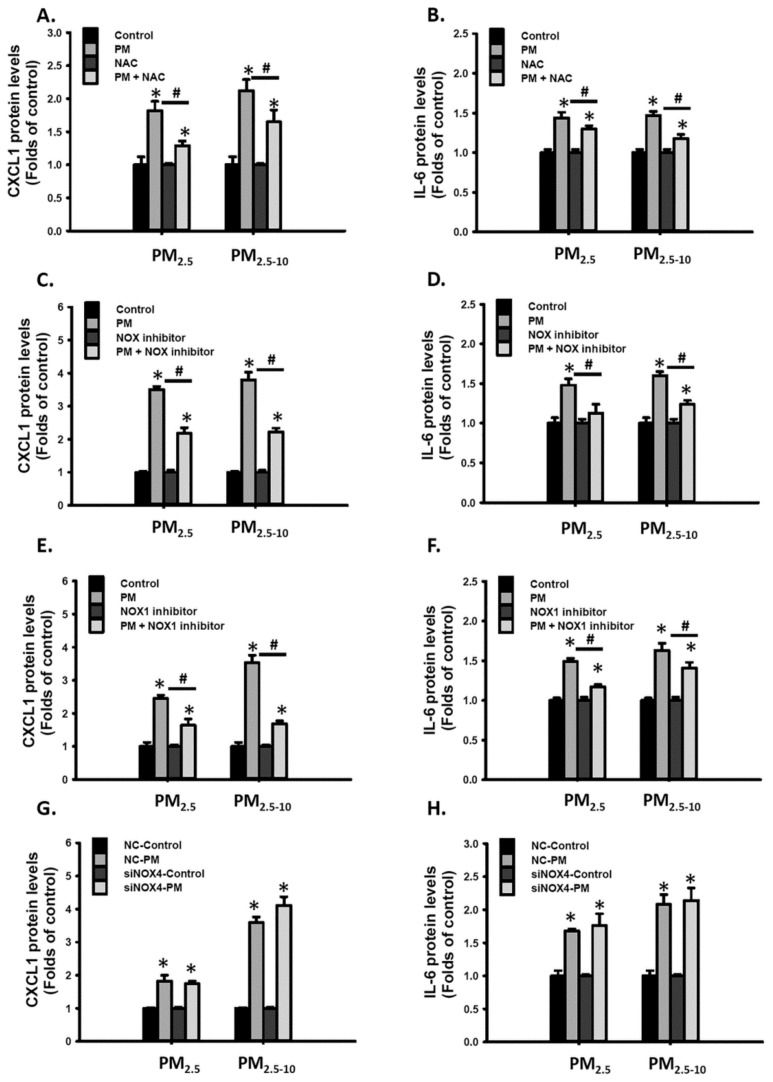
PM_2.5_ and PM_2.5-10_ increased inflammatory cytokine secretion partially through the NOX1-ROS signaling pathway in MVSMCs. MVSMCs were treated with d_2_H_2_O or 25 μg/mL PM_2.5_ or PM_2.5-10_ with and without 10 μM NAC for 48 h. (**A**) CXCL1 and (**B**) IL-6 protein concentrations were measured in the media. MVSMCs were treated with d_2_H_2_O or 25 μg/mL PM_2.5_ or PM_2.5-10_ with and without 10 μM NOX inhibitor for 48 h. (**C**) CXCL1 and (**D**) IL-6 protein concentrations were measured in the media. MVSMCs were treated with d_2_H_2_O or 25 μg/mL PM_2.5_ or PM_2.5-10_ with and without 10 μM NOX1 inhibitor for 48 h. (**E**) CXCL1 and (**F**) IL-6 protein concentrations were measured in the media. NC-MVSMCs and siNOX4-MVSMCs were treated with d_2_H_2_O or 25 μg/mL PM_2.5_ or PM_2.5-10_ for 48 h. (**G**) CXCL1 and (**H**) IL-6 protein concentrations were measured in the media. The results are presented as the mean ± SD for the three or three independent experiments. * *p* < 0.05, compared with vehicle-treated cells. # *p* < 0.05, compared with inhibitor-treated cells. Con: d_2_H_2_O treatment as control; NC: negative control for siRNA.

Furthermore, we investigated whether ROS are involved in PM-induced CXCL1 and IL-6 expressions. Cotreatment with NAC, NOX inhibitor, or NOX1 inhibitor partially blocked PM_2.5-10_- and PM_2.5_-increased CXCL1 and IL-6 secretions in MVSMCs (Figure 5A–F). By contrast, reducing NOX-4 expression did not inhibit PM_2.5-10_- and PM_2.5_-increased CXCL1 and IL-6 secretions (Figure 5G,H). These results indicate that PM_2.5-10_- and PM_2.5_ induced proinflammatory cytokine expression partially through the NOX1-ROS signaling pathway in MVSMCs.

### 3.6. Ambient PM_2.5-10_ and PM_2.5_ Induced VSMC Phenotypic Changes Through the NOX1/ROS/NF-κB Signaling Pathway

**Figure 6:**
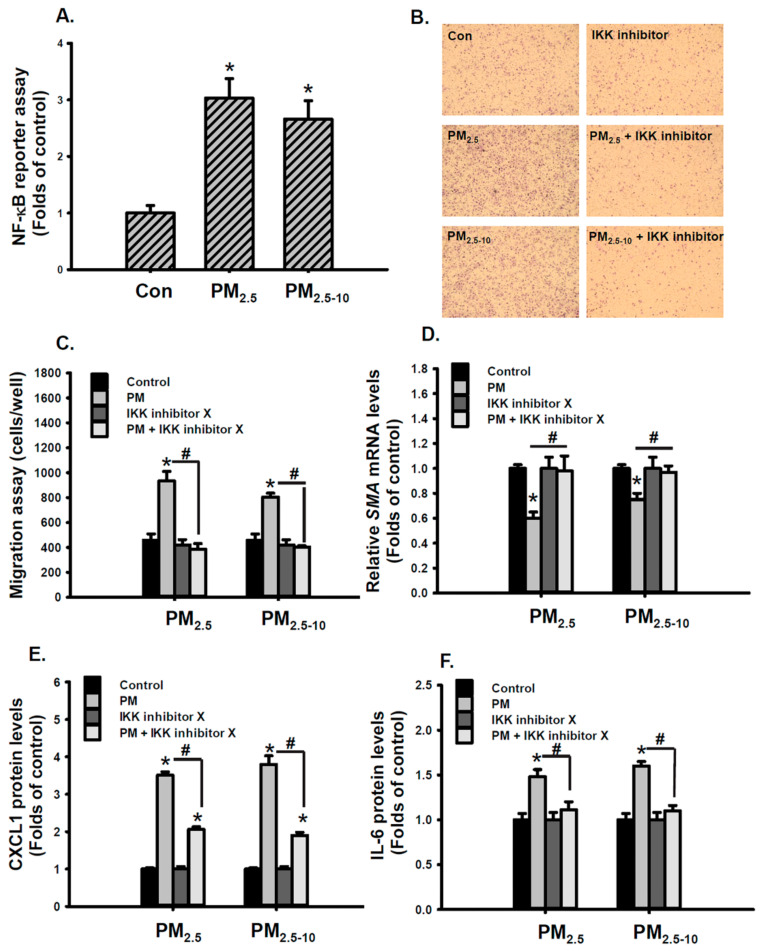
PM_2.5_ and PM_2.5-10_ induced cell phenotypic changes through the NF-κB signaling pathway in MVSMCs. MVSMCs were treated with d_2_H_2_O or 25 μg/mL PM_2.5_ or PM_2.5-10_ for 48 h. (**A**) NF-κB reporter activity was measured. MVSMCs were treated with d_2_H_2_O or 25 μg/mL PM_2.5_ or PM_2.5-10_ with and without 10 μM IKK inhibitor for 48 h. (**B**) and (**C**) Migration abilities of MVSMCs were detected and quantified using MetaMorph software. (**D**) The relative SMA mRNA levels were determined using a real-time PCR assay. (**E**) and (**F**) CXCL1 and IL-6 protein concentrations in the media were determined using the enzyme-linked immunosorbent assay. The results are presented as the mean ± SD for three or three independent experiments. * *p* < 0.05, compared with vehicle-treated cells. # *p* < 0.05, compared with IKK inhibitor-treated cells. Con: d_2_H_2_O treatment as control.

NF-κB plays a key role in the development of atherosclerosis [36] and the regulation of VSMC migration [37,38]. We further investigated whether NF-κB is involved in PM-induced VSMC phenotypic changes. PM_2.5-10_ and PM_2.5_ significantly increased NF-κB reporter activity (Figure 6A). NF-kB activation was also confirmed by the increased accumulation of phosphorylated p65 in the nuclei of MVSMCs in lung tissues of mice exposed to PM_2.5-10_ for 2 weeks (Figure S5). Cotreatment with IKK inhibitor X, an NF-κB inhibitor, completely blocked PM_2.5-10_- and PM_2.5_-enhanced migratory ability (Figure 6B,C). IKK inhibitor X also rescued PM_2.5-10_- and PM_2.5_-reduced SMA mRNA levels (Figure 6D). Similarly, IKK inhibitor X prevented PM_2.5-10_- and PM_2.5_-induced CXCL1 and IL-6 secretions (Figure 6E,F). These results indicate that NF-κB plays a critical role in PM_2.5-10_- and PM_2.5_-induced VSMC phenotypic changes.

**Figure 7:**
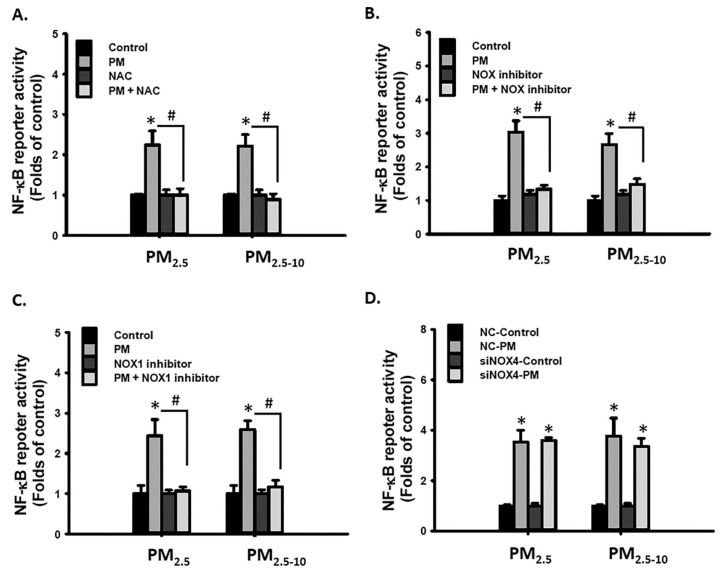
PM_2.5_ and PM_2.5-10_ activated the NOX1/ROS/NF-κB signaling pathway in MVSMCs. MVSMCs were treated with d_2_H_2_O or 25 μg/mL PM_2.5_ or PM_2.5-10_ with and without (**A**) 10 μM NAC, (**B**) 10 μM NOX inhibitor, and (**C**) 10 μM NOX1 inhibitor. (**D**) MVSMCs were transfected with NC-RNA or siNOX4 RNA. Transfected MVSMCs were treated with d_2_H_2_O or 25 μg/mL PM_2.5_ or PM_2.5-10._ The NF-κB reporter activity was measured after treatment for 48 h. The results are presented as the mean ± SD for four independent experiments. * *p* < 0.05, compared with vehicle-treated cells. # *p* < 0.05, compared with inhibitor-treated cells. Con: d_2_H_2_O treatment as control; NC: negative control for siRNA.

Studies have demonstrated that PM exposure activated NF-κB via ROS in human bronchial epithelial cells [39] and human umbilical vein endothelial cells [40]. In the current study, cotreatment with NAC, NOX inhibitor, or NOX1 inhibitor blocked PM_2.5-10_- and PM_2.5_- increased NF-κB reporter activity (Figure 7A–C), but reducing NOX4 expression did not exhibit similar effects (Figure 7D). These results reveal that the NOX1/ROS/ NF-κB signaling pathway was involved in the mechanism of PM_2.5-10_- and PM_2.5_-induced migration and proinflammatory cytokine secretion in MVSMCs.

### 3.7. Polyphenolic Compounds Prevented PM_2.5_-Induced ROS, NF-κB Activation, Cell Migration, and Proinflammatory Cytokine Secretion in MVSMCs

**Figure 8:**
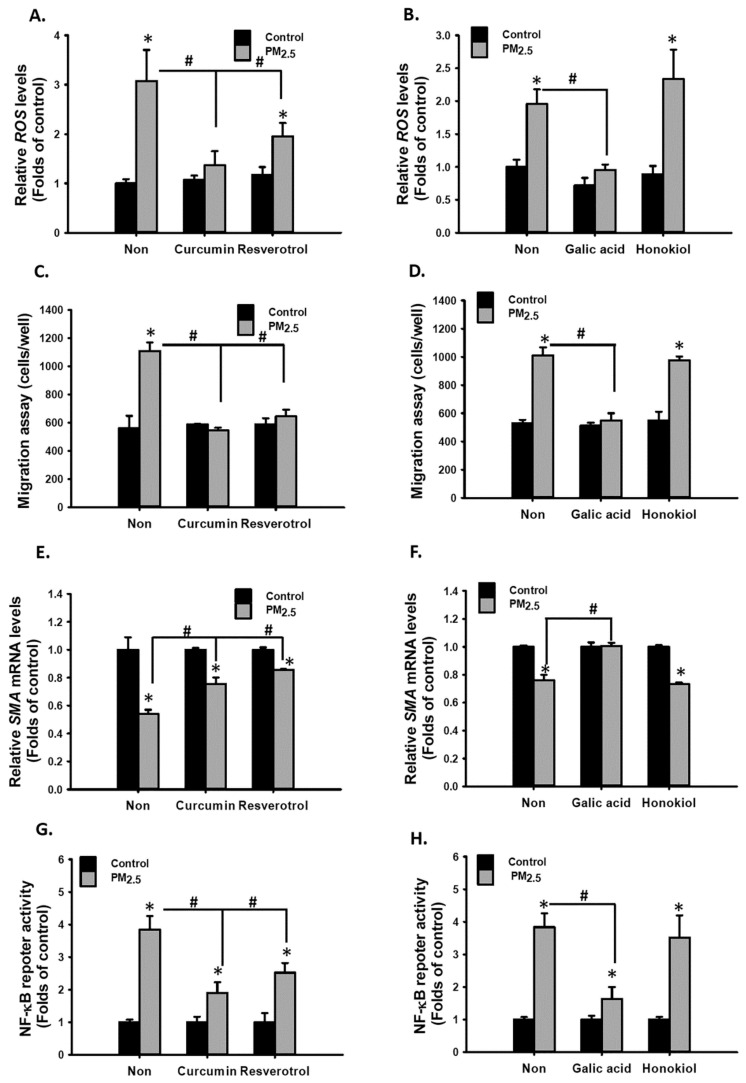
Polyphenolic compounds prevented PM_2.5_-induced oxidative stress, phenotypic changes, and NF-κB activation in MVSMCs. MVSMCs were treated with d_2_H_2_O or 12.5 μg/mL PM_2.5_ or PM_2.5-10_ with and without 5 μM curcumin, resveratrol, GA, or honokiol for 48 h. The following parameters were determined: (**A**,**B**) relative ROS levels; (**C**,**D**) cell migration ability; (**E**,**F**) relative SMA mRNA levels; (**G**,**H**) NF-κB reporter activity. The results are presented as the mean ± SD for the three independent experiments. * *p* < 0.05, compared with d_2_H_2_O -treated cells. # *p* < 0.05, compared with herbal compound-treated cells.

**Figure 9:**
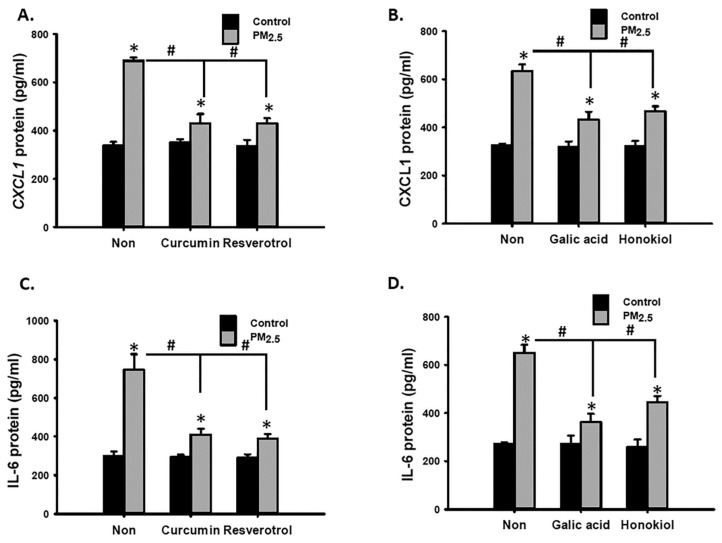
Polyphenolic compounds prevented PM_2.5_-induced inflammatory cytokine secretion in MVSMCs. MVSMCs were treated with d_2_H_2_O or 25 μg/mL PM_2.5_ or PM_2.5-10_ with and without 5 μM curcumin, resveratrol, GA, or honokiol. (**A**,**B**) CXCL1 protein concentrations in the media were quantified. (**C**,**D**) IL-6 protein concentrations in the media were quantified. The results are presented as the mean ± SD for three independent experiments. * *p* < 0.05, compared with d_2_H_2_O-treated cells. # *p* < 0.05, compared with herbal compound-treated cells.

ROS and inflammation are involved in cardiovascular diseases [41]. Antioxidative and anti-inflammatory polyphenolic compounds may be useful for preventing PM-induced VSMC phenotypic changes. Curcumin [42], resveratrol [43], and GA [44] are reported to exhibit antioxidative and anti-inflammatory characteristics. Nevertheless, honokiol is exclusively anti-inflammatory [45]. First, cotreatment with curcumin, resveratrol, and GA completely blocked PM_2.5_-increased ROS levels, but honokiol did not prevent an increase in ROS levels (Figure 8A,B). In addition, curcumin, resveratrol, and GA prevented PM_2.5_-enhanced migratory ability (Figure 8C,D), rescued PM_2.5_-reduced SMA expression (Figure 8E,F), and inhibited PM_2.5_-induced NF-κB reporter activity (Figure 8G,H). However, honokiol did not exhibit similar effects (Figure 8D,F,H). Nevertheless, the four polyphenolic compounds were able to prevent PM_2.5_-induced CXCL-1 and IL-6 secretions in MVSMCs (Figure 9A–D). These results suggest that curcumin, resveratrol, and GA prevented PM_2.5_-induced migration and cytokine secretion through the ROS-dependent NF-κB signaling pathway. However, honokiol prevented PM_2.5_-induced proinflammatory cytokine secretion through a mechanism independent of ROS/NF-κB pathway.

## 4. Discussion

**Figure 10:**
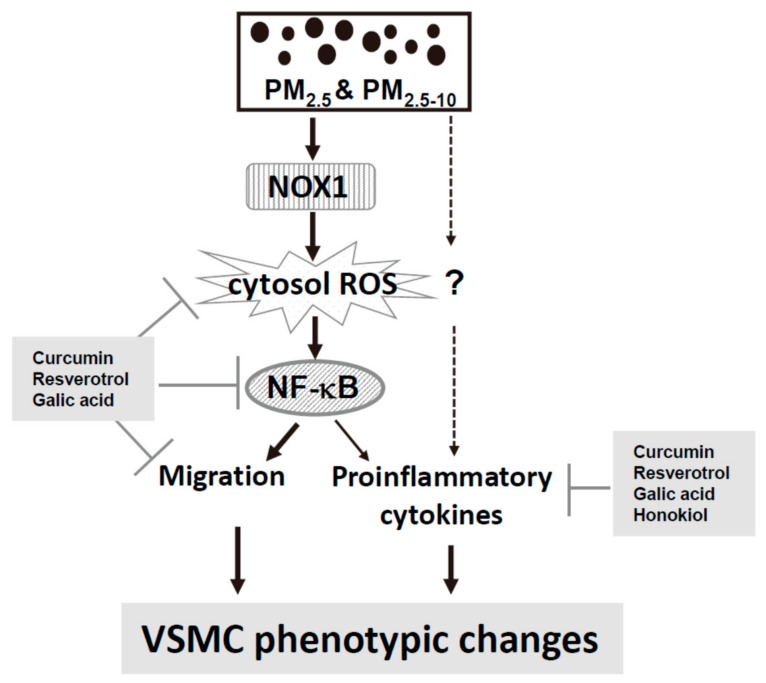
Summary of the mechanisms through which phenotypic changes in MVSMCs was induced by PM_2.5_ and PM_2.5-10_ and prevented by polyphenolic compounds.

Both epidemiological and animal studies suggested that vascular cells may be a major target for ambient PM. Furthermore, our previous studies have demonstrated that PM exposure disturbed VSMC function [13,18], but the mechanisms were not elucidated. The present study indicates that exposure to ambient PM_2.5_ and PM_2.5-10_ caused VSMC phenotypic changes, including increased migration ability and proinflammatory cytokines secretion, through the NOX1/ROS/NF-κB signaling pathway (Figure 10). A mechanism-based preventive approach with polyphenolic compounds was also suggested. These findings provide critical new insights into the mechanisms and prevention of PM-induced vascular diseases.

The NOX family has seven members, and NOX1 and NOX4 are both expressed in human and mouse VSMCs [46]. Chang et al. [47] demonstrated that PM and cigarette smoke induced ROS generation via NOX1, but not NOX4 in rat VSMC. Consistently, we also observed that PM induced ROS generation via NOX-1, but not NOX4 in MVSMC. However, PM failed to increase NOX-1 mRNA or protein levels in MVSMC (Figure S6). Some studies indicated that NOX1 and NOX4 produce different ROS and their activities are differently regulated. NOX1 produces primarily O_2_^•−^, but NOX4 directly produce H_2_O_2_ [48]. While NOX1 associates with the membrane-bound subunit, p22phox, and interacts with other cytosolic subunits [48], NOX4 only associates with p22phox [49]. It is possible that PM might regulate NOX1 activity via modulation of NOX1 associated cytosolic subunits. However, whether PM increases NOX1 activity in VSMCs remains unclear, and the possible mechanisms deserve further investigation.

NOX-derived ROS plays a crucial role in VSMC migration, proliferation, and inflammation [50]. Treatment with a NOX inhibitor significantly reduced the formation of angioplasty-induced neointimal hyperplasia in rats, which depends on the inhibition of VSMC migration and proliferation [51]. Some studies showed that NOX1 and NOX4 separately involved in mechanisms of different growth factors-mediated VSMC functions. For example, NOX1 is required for PDGF and basic fibroblast growth factor mediated VSMC migration [35,52]. NOX4 is required for insulin-like growth factor-mediated VSMC migration [53]. In the present study, we observed that NOX1-derived ROS contributed to PM-induced migration, but not NOX4. Understanding the role of NOX1 can assist in the development of preventive strategies for PM-induced vascular diseases.

Migration is a complex process which involves many elements, including cellular redox balance, regulation of the actin cytoskeleton and extracellular molecules. Numerous extracellular molecules such as growth factors, cytokines, and extracellular matrix components may promote SMC migration through different mechanisms [52,54,55]. For example, angiotensin II induced cell migration by activating the NOX/ROS/NF-κB/IL-6/MMP-9 pathway in human aortic SMCs [56] or by activating the c-Src/RhoA pathway in rat VSMCs [57]. Moreover, TNF-α mediated VSMC migration requires NF-κB activation [38]. The TLR2/NF-κB/NFATc1 signaling pathway activated by PDGF [58] and the ROS/NF-κB pathway activated by vascular endothelial growth factor [37] are both involved in growth factors–stimulated VSMC migration. In the present study, we found that PM induced VSMC migration through the NOX1/ROS-dependent NF-κB signaling pathway, which was also partially involved in inducing pro-inflammatory cytokine secretion. The involvement of growth factors in PM-induced VSMC phenotypic changes deserves further investigation. Although proinflammatory cytokines (such as IL-6) were reported to increase VMSC migration [59], our results suggest that the PM-increased cytokine secretion and cell migration effects observed in the VSMCs occurred independently. The PM-increased IL-6 concentration (approximately 20 pg/mL) might be too low, compared with the 1 ng/mL concentration that effectively promoted VSMC migration [60].

IL-6 plays roles in acute inflammation and adaptive tissue remodeling [61]. At the early phase of tissue injury, IL-6 is immediately secreted, induces the release of proinflammatory cytokines and induces chemotaxis of leukocytes to the injured tissues. Later, IL-6 switches macrophage polarization and promotes the secretion of mediators for tissues repair and remodeling. Then, IL-6 levels will return to normal during tissue remodeling. In our present animal study, exposure to PM injured lung tissues, increased IL-6 secretion and caused pulmonary inflammation within 2 weeks. The injured tissues were gradually repaired, as indicated by the decline of LDH activity and IL-6 concentrations in BALF at week 4 and 8. Meanwhile, the increase in collagen fiber deposition and the medial thickening indicated vascular remodeling at week 4 and 8.

Atherosclerosis is one of the major vascular diseases induced by PM_2.5_. More recently, Liang et al. [62] indicated that vascular endothelial injury plays a critical role in PM_2.5_-trigered atherosclerosis. Endothelial cells consist of a single layer of the tunica intima. First, PM_2.5_ disturbed vascular endothelial cells functions, including increased endothelial permeability, declined vascular tone and vascular reparative capability. Activated endothelial cells promoted the monocytes recruited and the maturation of monocytes into macrophages. Macrophages accumulated oxidized lipids, and finally promoted foam cell formation. On the other hand, VSMCs in the tunica media may respond to the signals of injured endothelial cells and switch to highly migratory and proliferative phenotypes [63]. Liang et al. [62] pointed out that oxidative stress induced by PM_2.5_ can increase VSMCs proliferation. Our present study further demonstrated that oxidative stress induced by PM_2.5_ increased VMSCs migration. Some studies reported that VSMCs were present in the atherosclerotic plagues and suggested that VMSCs migrate from the tunica media into the tunica intima during the development of atherosclerosis [63,64]. Thus, it is possible that the increase in VSMCs migration may also play a role in PM_2.5_-trigered atherosclerosis.

Certain herbal compounds have been reported to prevent PM-induced oxidative stress and inflammation in endothelial cells, bronchial epithelial cells, and lung or heart tissue [65,66,67]. Several herbal compounds have been used to inhibit NOX activity, including resveratrol [65], and epigallocatechin-3-gallate [67], but the inhibitory effects are not specific to the NOX family. In the present study, we evaluated the preventive effects of polyphenolic compounds in PM-induced vascular phenotypic changes. At the dosage of 5 μM, three polyphenolic compounds, including curcumin, resveratrol, and GA, exhibit antioxidative and anti-inflammatory properties, but 5 μM honokiol is exclusively anti-inflammatory. As expected, the four polyphenolic compounds prevented PM-increased cytokines secretion. However, treatment with 5 μM honokiol failed to prevent PM-induced ROS, NF-κB activity or migration in MVSMC. It appears that the antioxidant properties of the compounds were required to prevent PM-induced VSMC migration. Some studies report that 10 μM honokiol reduced ROS levels and prevented inflammation in cells [68,69]. Our results suggested that honokiol at the dosage of 5 μM might prevented PM-induced cytokines secretion via NF-κB independent pathways, for example the the PI3K/Akt pathway [70].

Toxicological studies have suggested that ambient PM_2.5-10_ has considerable inflammatory effects [71,72,73]. Heltand et al. [72] demonstrated that ambient PM_2.5-10_ was more potent in the induction of inflammatory cytokines than the corresponding ambient PM_2.5_ in rat lungs. Furthermore, PM_2.5-10_ was more toxic than PM_2.5_ in certain toxicological studies [74]. In the present study, we revealed that both PM_2.5-10_ and PM_2.5_ induced migration ability and cytokines secretion and activated the same pathways in VSMCs. The present study suggests that exposure to ambient PM_2.5-10_ and PM_2.5_ may induce vascular phenotypic changes in VSMCs through similar mechanisms.

## 5. Conclusions

The present study identified the NOX1/ROS/NF-κB pathway as the critical mechanism of ambient PM-induced VSMCs phenotypic changes, which included increased migration ability and proinflammatory cytokine secretion. Furthermore, polyphenolic compounds with antioxidative properties can prevent ambient PM-induced proinflammatory cytokines secretion and cell migration in VSMCs. However, honokiol prevented PM_2.5_-induced proinflammatory cytokines secretion through a mechanism independent of the ROS/NF-κB pathway.

## Data Availability

All the data presented in this study are included in the article.

## Supplementary Materials

The following are available online at https://www.mdpi.com/article/10.3390/antiox10050782/s1, Figure S1: PM_2.5_ induced vascular remodeling at 8 weeks in mouse lung; Figure S2: The cytotoxicity of PM_2.5_ and PM_2.5-10_ in HPASMCs, MPASMCs, and MVSMCs; Figure S3: Effect of PM_2.5_ and PM_2.5-10_ on cell phenotypic changes in HPASMCs, MPASMCs, and MVSMCs; Figure S4: The cytotoxicity of polyphenolic compounds in MVSMCs. Figure S5: Immunohistochemical staining for NF-κB subunit phospho-p65 in small arteries of mouse lung. Figure S6: Effects of PM on the NOX1 mRNA and protein levels in MVSMCs.

## References

[1] Hayes R.B., Lim C., Zhang Y., Cromar K., Shao Y., Reynolds H.R., Silverman D.T., Jones R.R., Park Y., Jerrett M. (2019). PM2.5 air pollution and cause-specific cardiovascular disease mortality. Int. J. Epidemiol..

[2] Brook R.D., Rajagopalan S., Pope C.A., Brook J.R., Bhatnagar A., Diez-Roux A.V., Holguin F., Hong Y., Luepker R.V., Mittleman M.A. (2010). Particulate matter air pollution and cardiovascular disease: An update to the scientific statement from the American Heart Association. Circulation.

[3] Vaduganathan M., De Palma G., Manerba A., Goldoni M., Triggiani M., Apostoli P., Dei Cas L., Nodari S. (2016). Risk of Cardiovascular Hospitalizations from Exposure to Coarse Particulate Matter (PM10) B elow the European Union Safety Threshold. Am. J. Cardiol..

[4] Polak J.F. (2009). Carotid intima-media thickness: An early marker of cardiovascular disease. Ultrasound Q..

[5] Bauer M., Moebus S., Mohlenkamp S., Dragano N., Nonnemacher M., Fuchsluger M., Kessler C., Jakobs H., Memmesheimer M., Erbel R. (2010). Urban particulate matter air pollution is associated with subclinical atherosclerosis: Results from the HNR (Heinz Nixdorf Recall) study. J. Am. Coll. Cardiol..

[6] Kunzli N., Jerrett M., Mack W.J., Beckerman B., LaBree L., Gilliland F., Thomas D., Peters J., Hodis H.N. (2005). Ambient air pollution and atherosclerosis in Los Angeles. Environ. Health Perspect..

[7] Lenters V., Uiterwaal C.S., Beelen R., Bots M.L., Fischer P., Brunekreef B., Hoek G. (2010). Long-term exposure to air pollution and vascular damage in young adults. Epidemiology.

[8] Su T.C., Hwang J.J., Shen Y.C., Chan C.C. (2015). Carotid Intima-Media Thickness and Long-Term Exposure to Traffic-Related Air Pollution in Middle-Aged Residents of Taiwan: A Cross-Sectional Study. Environ. Health Perspect..

[9] Zhang J., Cai L., Gui Z., Wang S., Zeng X., Lai L., Lv Y., Tan K., Wang H., Huang C. (2020). Air pollution-associated blood pressure may be modified by diet among children in Guangzhou, China. J. Hypertens..

[10] Chen S.Y., Wu C.F., Lee J.H., Hoffmann B., Peters A., Brunekreef B., Chu D.C., Chan C.C. (2015). Associations between Long-Term Air Pollutant Exposures and Blood Pressure in Elderly Residents of Taipei City: A Cross-Sectional Study. Environ. Health Perspect..

[11] Calderon-Garciduenas L., Vincent R., Mora-Tiscareno A., Franco-Lira M., Henriquez-Roldan C., Barragan-Mejia G., Garrido-Garcia L., Camacho-Reyes L., Valencia-Salazar G., Paredes R. (2007). Elevated plasma endothelin-1 and pulmonary arterial pressure in children exposed to air pollution. Environ. Health Perspect..

[12] Sofianopoulou E., Kaptoge S., Graf S., Hadinnapola C., Treacy C.M., Church C., Coghlan G., Gibbs J.S.R., Haimel M., Howard L.S. (2019). Traffic exposures, air pollution and outcomes in pulmonary arterial hypertension: A UK cohort study analysis. Eur. Respir. J..

[13] Ho C.C., Tsai M.H., Chen Y.C., Kuo C.C., Lin P. (2019). Persistent elevation of blood pressure by ambient coarse particulate matter after recovery from pulmonary inflammation in mice. Environ. Toxicol..

[14] Xiao X., Yao T., Du S., Zhang J., Huang T., Lei Y., Cao L., Shen Z., Cao Y. (2020). Age differences in the pulmonary and vascular pathophysiologic processes after long-term real-time exposure to particulate matter in rats. Chemosphere.

[15] Kawai-Kowase K., Owens G.K. (2007). Multiple repressor pathways contribute to phenotypic switching of vascular smooth muscle cells. Am. J. Physiol. Cell Physiol..

[16] Xu C.B., Lei Y., Chen Q., Pehrson C., Larsson L., Edvinsson L. (2010). Cigarette smoke extracts promote vascular smooth muscle cell proliferation and enhances contractile responses in the vasculature and airway. Basic Clin. Pharmacol. Toxicol..

[17] Starke R.M., Ali M.S., Jabbour P.M., Tjoumakaris S.I., Gonzalez F., Hasan D.M., Rosenwasser R.H., Owens G.K., Koch W.J., Dumont A.S. (2013). Cigarette smoke modulates vascular smooth muscle phenotype: Implications for carotid and cerebrovascular disease. PLoS ONE.

[18] Ho C.C., Wu W.T., Chen Y.C., Liou S.H., Yet S.F., Lee C.H., Tsai H.T., Weng C.Y., Tsai M.H., Lin P. (2019). Identification of osteopontin as a biomarker of human exposure to fine particulate matter. Environ. Pollut..

[19] Steven S., Frenis K., Oelze M., Kalinovic S., Kuntic M., Bayo Jimenez M.T., Vujacic-Mirski K., Helmstadter J., Kroller-Schon S., Munzel T. (2019). Vascular Inflammation and Oxidative Stress: Major Triggers for Cardiovascular Disease. Oxid. Med. Cell. Longev..

[20] Senoner T., Dichtl W. (2019). Oxidative Stress in Cardiovascular Diseases: Still a Therapeutic Target?. Nutrients.

[21] Ho C.C., Chen Y.C., Yet S.F., Weng C.Y., Tsai H.T., Hsu J.F., Lin P. (2020). Identification of ambient fine particulate matter components related to vascular dysfunction by analyzing spatiotemporal variations. Sci. Total Environ..

[22] Friedman M. (2007). Overview of antibacterial, antitoxin, antiviral, and antifungal activities of tea flavonoids and teas. Mol. Nutr. Food Res..

[23] Azhdari M., Karandish M., Mansoori A. (2019). Metabolic benefits of curcumin supplementation in patients with metabolic syndrome: A systematic review and meta-analysis of randomized controlled trials. Phytother. Res..

[24] Asgary S., Karimi R., Momtaz S., Naseri R., Farzaei M.H. (2019). Effect of resveratrol on metabolic syndrome components: A systematic review and meta-analysis. Rev. Endocr. Metab. Disord..

[25] Choubey S., Varughese L.R., Kumar V., Beniwal V. (2015). Medicinal importance of gallic acid and its ester derivatives: A patent review. Pharm. Pat. Anal..

[26] Jin L., Piao Z.H., Sun S., Liu B., Kim G.R., Seok Y.M., Lin M.Q., Ryu Y., Choi S.Y., Kee H.J. (2017). Gallic Acid Reduces Blood Pressure and Attenuates Oxidative Stress and Cardiac Hypertrophy in Spontaneously Hypertensive Rats. Sci. Rep..

[27] He F., Liao B., Pu J., Li C., Zheng M., Huang L., Zhou Y., Zhao D., Li B., Ran P. (2017). Exposure to Ambient Particulate Matter Induced COPD in a Rat Model and a Description of the Underlying Mechanism. Sci. Rep..

[28] Wang C.K., Chang L.W., Chang H., Yang C.H., Tsai M.H., Tsai H.T., Lin P. (2010). Pulmonary changes induced by trans,trans-2,4-decadienal, a component of cooking oil fumes. Eur. Respir. J..

[29] Lin P., Chang H., Ho W.L., Wu M.H., Su J.M. (2003). Association of aryl hydrocarbon receptor and cytochrome P4501B1 expressions in human non-small cell lung cancers. Lung Cancer.

[30] Gunther S., Alexander R.W., Atkinson W.J., Gimbrone M.A. (1982). Functional angiotensin II receptors in cultured vascular smooth muscle cells. J. Cell Biol..

[31] Ho C.C., Luo Y.H., Chuang T.H., Yang C.S., Ling Y.C., Lin P. (2013). Quantum dots induced monocyte chemotactic protein-1 expression via MyD88-dependent Toll-like receptor signaling pathways in macrophages. Toxicology.

[32] San Martin A., Griendling K.K. (2010). Redox control of vascular smooth muscle migration. Antioxid. Redox Signal..

[33] Simkhovich B.Z., Kleinman M.T., Kloner R.A. (2008). Air pollution and cardiovascular injury epidemiology, toxicology, and mechanisms. J. Am. Coll. Cardiol..

[34] Burtenshaw D., Hakimjavadi R., Redmond E.M., Cahill P.A. (2017). Nox, Reactive Oxygen Species and Regulation of Vascular Cell Fate. Antioxidants.

[35] Lee M.Y., San Martin A., Mehta P.K., Dikalova A.E., Garrido A.M., Datla S.R., Lyons E., Krause K.H., Banfi B., Lambeth J.D. (2009). Mechanisms of vascular smooth muscle NADPH oxidase 1 (Nox1) contribution to injury-induced neointimal formation. Arter. Thromb. Vasc. Biol..

[36] Landry D.B., Couper L.L., Bryant S.R., Lindner V. (1997). Activation of the NF-kappa B and I kappa B system in smooth muscle cells after rat arterial injury. Induction of vascular cell adhesion molecule-1 and monocyte chemoattractant protein-1. Am. J. Pathol..

[37] Wang Z., Castresana M.R., Newman W.H. (2001). Reactive oxygen and NF-kappaB in VEGF-induced migration of human vascular smooth muscle cells. Biochem. Biophys. Res. Commun..

[38] Wang Z., Castresana M.R., Newman W.H. (2001). NF-kappaB is required for TNF-alpha-directed smooth muscle cell migration. FEBS Lett..

[39] Wang J., Huang J., Wang L., Chen C., Yang D., Jin M., Bai C., Song Y. (2017). Urban particulate matter triggers lung inflammation via the ROS-MAPK-NF-kappaB signaling pathway. J. Thorac. Dis..

[40] Rui W., Guan L., Zhang F., Zhang W., Ding W. (2016). PM2.5-induced oxidative stress increases adhesion molecules expression in human endothelial cells through the ERK/AKT/NF-kappaB-dependent pathway. J. Appl. Toxicol..

[41] Lakshmi S.V., Padmaja G., Kuppusamy P., Kutala V.K. (2009). Oxidative stress in cardiovascular disease. Indian J. Biochem. Biophys..

[42] Pari L., Tewas D., Eckel J. (2008). Role of curcumin in health and disease. Arch. Physiol. Biochem..

[43] Hamza R.Z., El-Shenawy N.S. (2017). Anti-inflammatory and antioxidant role of resveratrol on nicotine-induced lung changes in male rats. Toxicol. Rep..

[44] Pal C., Bindu S., Dey S., Alam A., Goyal M., Iqbal M.S., Maity P., Adhikari S.S., Bandyopadhyay U. (2010). Gallic acid prevents nonsteroidal anti-inflammatory drug-induced gastropathy in rat by blocking oxidative stress and apoptosis. Free Radic. Biol. Med..

[45] Khalid S., Ullah M.Z., Khan A.U., Afridi R., Rasheed H., Khan A., Ali H., Kim Y.S., Khan S. (2018). Antihyperalgesic Properties of Honokiol in Inflammatory Pain Models by Targeting of NF-kappaB and Nrf2 Signaling. Front. Pharmacol..

[46] Clempus R.E., Griendling K.K. (2006). Reactive oxygen species signaling in vascular smooth muscle cells. Cardiovasc. Res..

[47] Chang K.H., Park J.M., Lee C.H., Kim B., Choi K.C., Choi S.J., Lee K., Lee M.Y. (2017). NADPH oxidase (NOX) 1 mediates cigarette smoke-induced superoxide generation in rat vascular smooth muscle cells. Toxicol. In Vitro.

[48] Schroder K., Weissmann N., Brandes R.P. (2017). Organizers and activators: Cytosolic Nox proteins impacting on vascular function. Free Radic. Biol. Med..

[49] Nguyen Dinh Cat A., Montezano A.C., Burger D., Touyz R.M. (2013). Angiotensin II, NADPH oxidase, and redox signaling in the vasculature. Antioxid. Redox Signal..

[50] Griendling K.K., Sorescu D., Ushio-Fukai M. (2000). NAD(P)H oxidase: Role in cardiovascular biology and disease. Circ. Res..

[51] Jacobson G.M., Dourron H.M., Liu J., Carretero O.A., Reddy D.J., Andrzejewski T., Pagano P.J. (2003). Novel NAD(P)H oxidase inhibitor suppresses angioplasty-induced superoxide and neointimal hyperplasia of rat carotid artery. Circ. Res..

[52] Cucina A., Borrelli V., Randone B., Coluccia P., Sapienza P., Cavallaro A. (2003). Vascular endothelial growth factor increases the migration and proliferation of smooth muscle cells through the mediation of growth factors released by endothelial cells. J. Surg. Res..

[53] Meng D., Lv D.D., Fang J. (2008). Insulin-like growth factor-I induces reactive oxygen species production and cell migration through Nox4 and Rac1 in vascular smooth muscle cells. Cardiovasc. Res..

[54] Gerthoffer W.T. (2007). Mechanisms of vascular smooth muscle cell migration. Circ. Res..

[55] Newby A.C. (2006). Matrix metalloproteinases regulate migration, proliferation, and death of vascular smooth muscle cells by degrading matrix and non-matrix substrates. Cardiovasc. Res..

[56] Tsai M.H., Lee C.W., Hsu L.F., Li S.Y., Chiang Y.C., Lee M.H., Chen C.H., Liang H.F., How J.M., Chang P.J. (2017). CO-releasing molecules CORM2 attenuates angiotensin II-induced human aortic smooth muscle cell migration through inhibition of ROS/IL-6 generation and matrix metalloproteinases-9 expression. Redox Biol..

[57] Montezano A.C., Callera G.E., Yogi A., He Y., Tostes R.C., He G., Schiffrin E.L., Touyz R.M. (2008). Aldosterone and angiotensin II synergistically stimulate migration in vascular smooth muscle cells through c-Src-regulated redox-sensitive RhoA pathways. Arterioscler. Thromb. Vasc. Biol..

[58] Cheng W., Yan K., Chen Y., Zhang W., Ji Z., Dang C. (2018). ABCA1 inhibits PDGF-induced proliferation and migration of rat airway smooth muscle cell through blocking TLR2/NF-kappaB/NFATc1 signaling. J. Cell. Biochem..

[59] Wang Z., Newman W.H. (2003). Smooth muscle cell migration stimulated by interleukin 6 is associated with cytoskeletal reorganization. J. Surg. Res..

[60] Newman W.H., Castresana M.R., Webb J.G., Wang Z. (2003). Cyclic AMP inhibits production of interleukin-6 and migration in human vascular smooth muscle cells. J. Surg. Res..

[61] Johnson B.Z., Stevenson A.W., Prele C.M., Fear M.W., Wood F.M. (2020). The Role of IL-6 in Skin Fibrosis and Cutaneous Wound Healing. Biomedicines.

[62] Liang S., Zhang J., Ning R., Du Z., Liu J., Batibawa J.W., Duan J., Sun Z. (2020). The critical role of endothelial function in fine particulate matter-induced atherosclerosis. Part. Fibre Toxicol..

[63] Owens G.K., Kumar M.S., Wamhoff B.R. (2004). Molecular regulation of vascular smooth muscle cell differentiation in development and disease. Physiol. Rev..

[64] Grootaert M.O.J., Bennett M.R. (2021). Vascular smooth muscle cells in atherosclerosis:Time for a reassessment. Cardiovasc. Res..

[65] Tang Y., Xu J., Qu W., Peng X., Xin P., Yang X., Ying C., Sun X., Hao L. (2012). Resveratrol reduces vascular cell senescence through attenuation of oxidative stress by SIRT1/NADPH oxidase-dependent mechanisms. J. Nutr. Biochem..

[66] Stolk J., Hiltermann T.J., Dijkman J.H., Verhoeven A.J. (1994). Characteristics of the inhibition of NADPH oxidase activation in neutrophils by apocynin, a methoxy-substituted catechol. Am. J. Respir. Cell Mol. Biol..

[67] Ahn H.Y., Kim C.H., Ha T.S. (2010). Epigallocatechin-3-gallate Regulates NADPH Oxidase Expression in Human Umbilical Vein Endothelial Cells. Korean J. Physiol. Pharmacol..

[68] Zhu X., Wang Z., Hu C., Li Z., Hu J. (2014). Honokiol suppresses TNF-alpha-induced migration and matrix metalloproteinase expression by blocking NF-kappaB activation via the ERK signaling pathway in rat aortic smooth muscle cells. Acta. Histochem..

[69] Chao L.K., Liao P.C., Ho C.L., Wang E.I., Chuang C.C., Chiu H.W., Hung L.B., Hua K.F. (2010). Anti-inflammatory bioactivities of honokiol through inhibition of protein kinase C, mitogen-activated protein kinase, and the NF-kappaB pathway to reduce LPS-induced TNFalpha and NO expression. J. Agric. Food Chem..

[70] Kim B.H., Cho J.Y. (2008). Anti-inflammatory effect of honokiol is mediated by PI3K/Akt pathway suppression. Acta Pharmacol. Sin..

[71] Becker S., Mundandhara S., Devlin R.B., Madden M. (2005). Regulation of cytokine production in human alveolar macrophages and airway epithelial cells in response to ambient air pollution particles: Further mechanistic studies. Toxicol. Appl. Pharmacol..

[72] Hetland R.B., Cassee F.R., Lag M., Refsnes M., Dybing E., Schwarze P.E. (2005). Cytokine release from alveolar macrophages exposed to ambient particulate matter: Heterogeneity in relation to size, city and season. Part. Fibre Toxicol..

[73] Schins R.P., Lightbody J.H., Borm P.J., Shi T., Donaldson K., Stone V. (2004). Inflammatory effects of coarse and fine particulate matter in relation to chemical and biological constituents. Toxicol. Appl. Pharmacol..

[74] Schwarze P.E., Ovrevik J., Lag M., Refsnes M., Nafstad P., Hetland R.B., Dybing E. (2006). Particulate matter properties and health effects: Consistency of epidemiological and toxicological studies. Hum. Exp. Toxicol..

