# Supplementary material for: Ambient Particulate Matter Induces Vascular Smooth Muscle Cell Phenotypic Changes via NOX1/ROS/NF-κB Dependent and Independent Pathways: Protective Effects of Polyphenols
Source: Antioxidants (Basel). 2021 May 14;10(5):782. doi: 10.3390/antiox10050782 (PMC8156007; doi:10.3390/antiox10050782)
Supplement: Supplementary file 1 [file antioxidants-10-00782-s001.zip › antioxidants-1208426-supplementary.pdf]

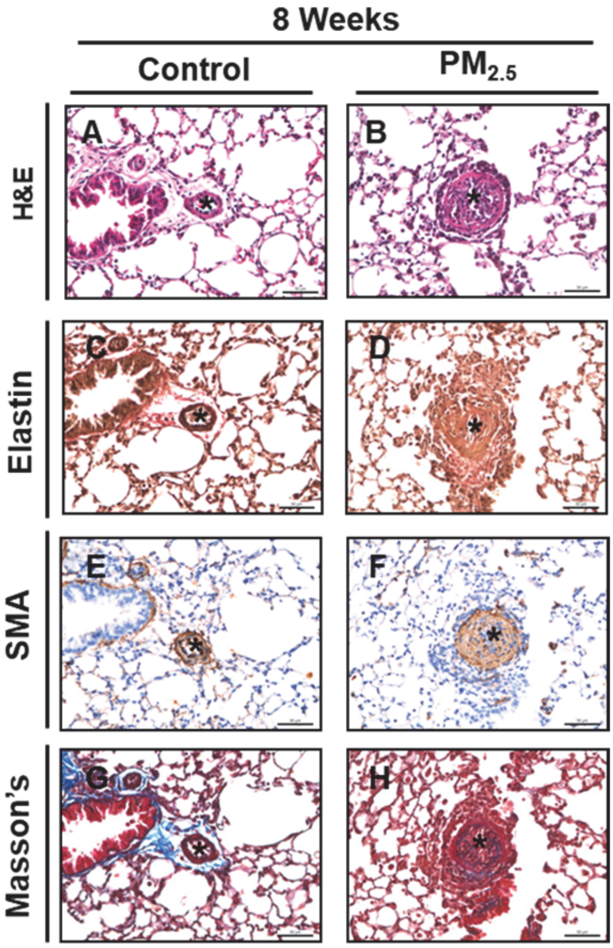

Figure S1. $PM_{2.5}$ induced vascular remodeling at 8 weeks in mouse lung.

Mice were aspirated with 25 µg $PM_{2.5}$ per mouse, and twice per week for 8 weeks. H&E; Verhoeff's staining; SMA staining; Masson's Trichome Staining. Star indicates small artery. Scale bar, 30 µm

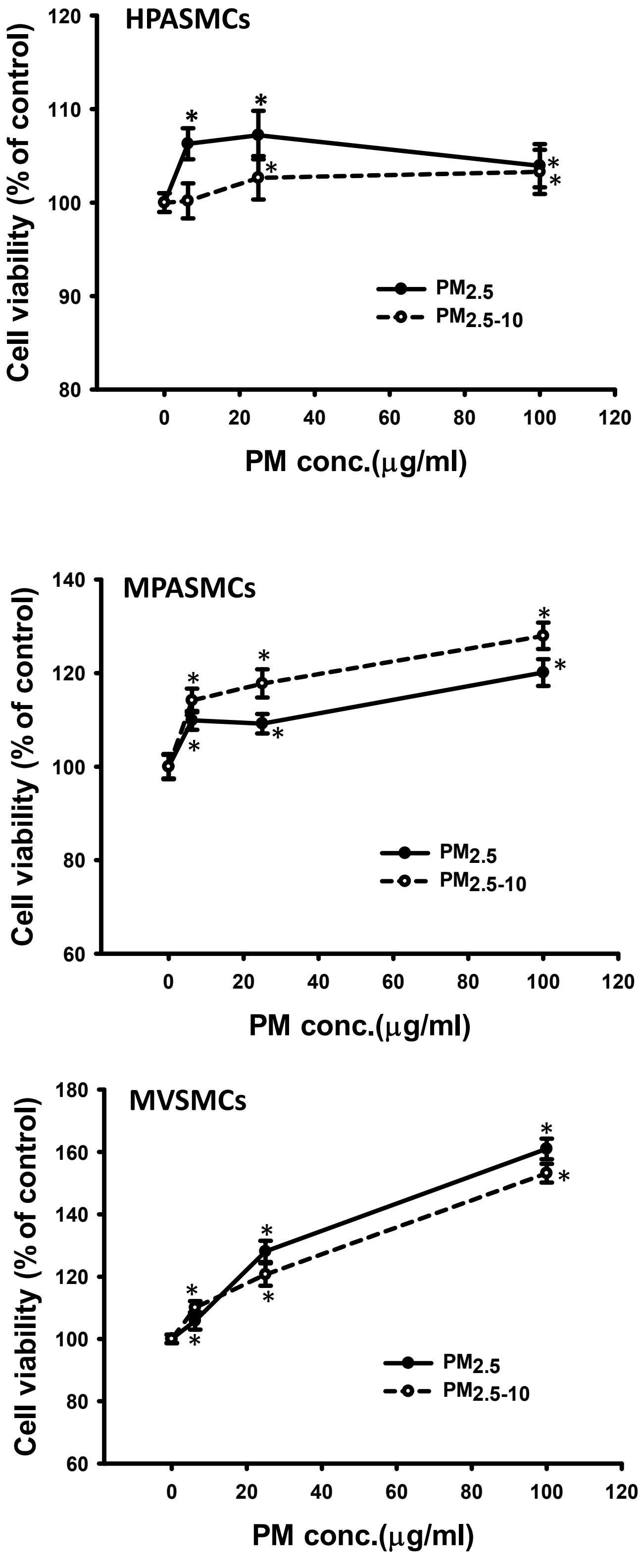

Figure S2. The cytotoxicity of $PM_{2.5}$ and $PM_{2.5\text{-}10}$ in HPASMCs, MPASMCs, and MVSMCs. HPASMCs, MPASMCs, and MVSMCs were treated with $PM_{2.5}$ and $PM_{2.5\text{-}10}$ for 48 h. The results are presented as the mean ± SD for eight independent experiments. *$p < 0.05$, compared with d2H2O-treated cells.

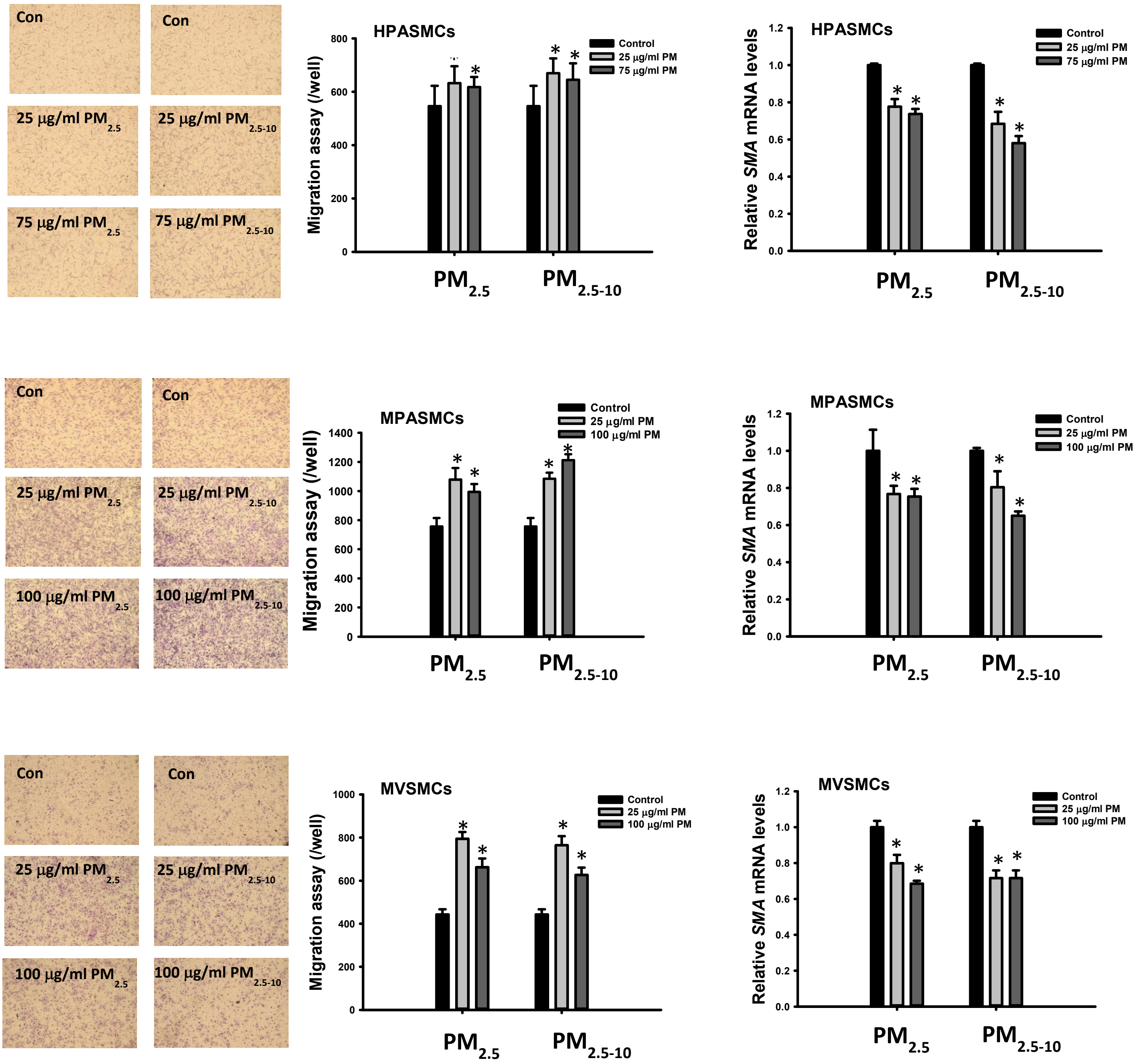

Figure S3. Effect of $PM_{2.5}$ and $PM_{2.5\text{-}10}$ on cell phenotypic changes in HPASMCs, MPASMCs, and MVSMCs. HPASMCs, MPASMCs, and MVSMCs were treated with $PM_{2.5}$ and $PM_{2.5\text{-}10}$ for 48 h. The results are presented as the mean ± SD for eight independent experiments. $*p < 0.05$, compared with d_2H_2O-treated cells.

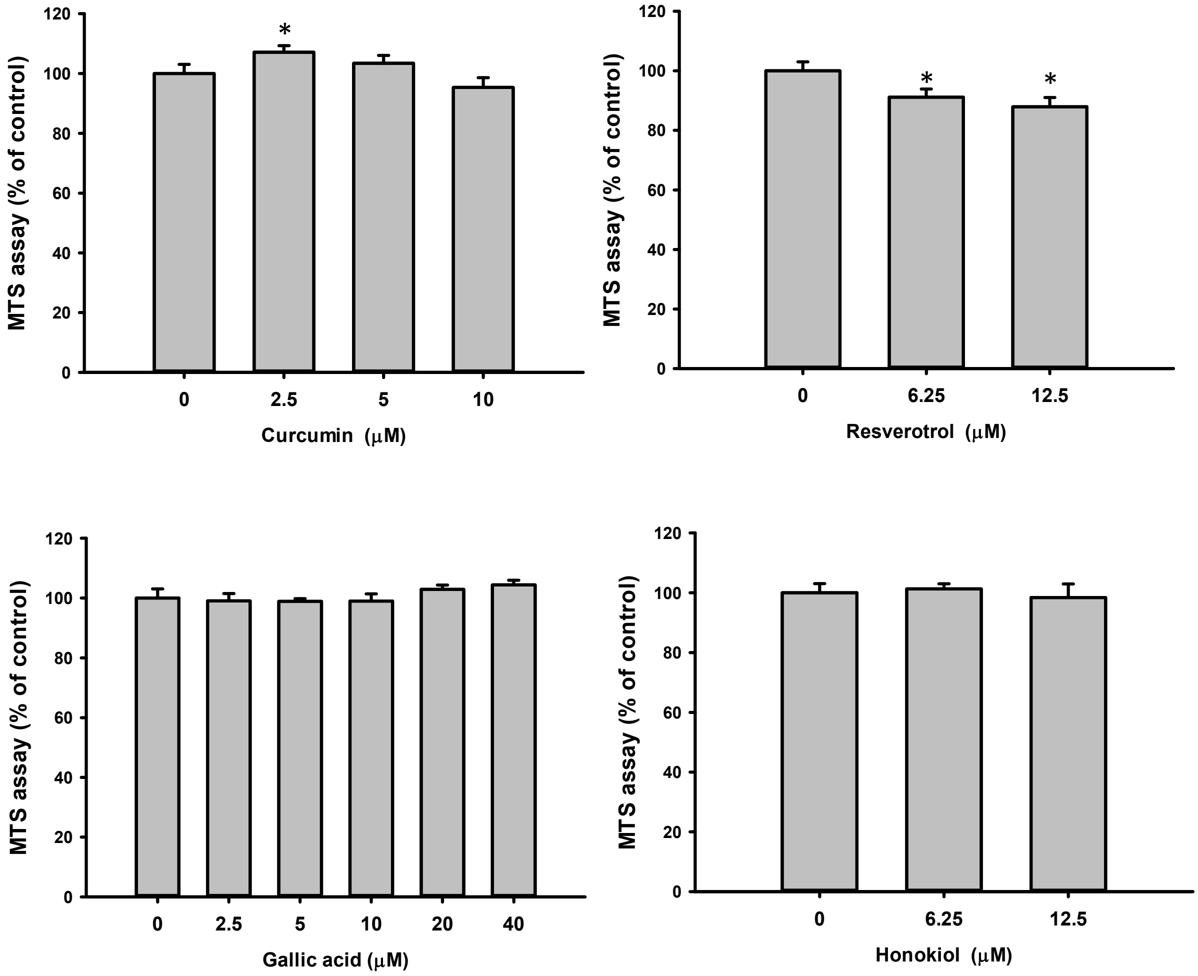

Figure S4. The cytotoxicity of polyphenolic compounds in MVSMCs.

MVSMCs were treated with polyphenolic compounds for 48 h. The results are presented as the mean ± SD for eight independent experiments. *p < 0.05, compared with d_2H_2O-treated cells.

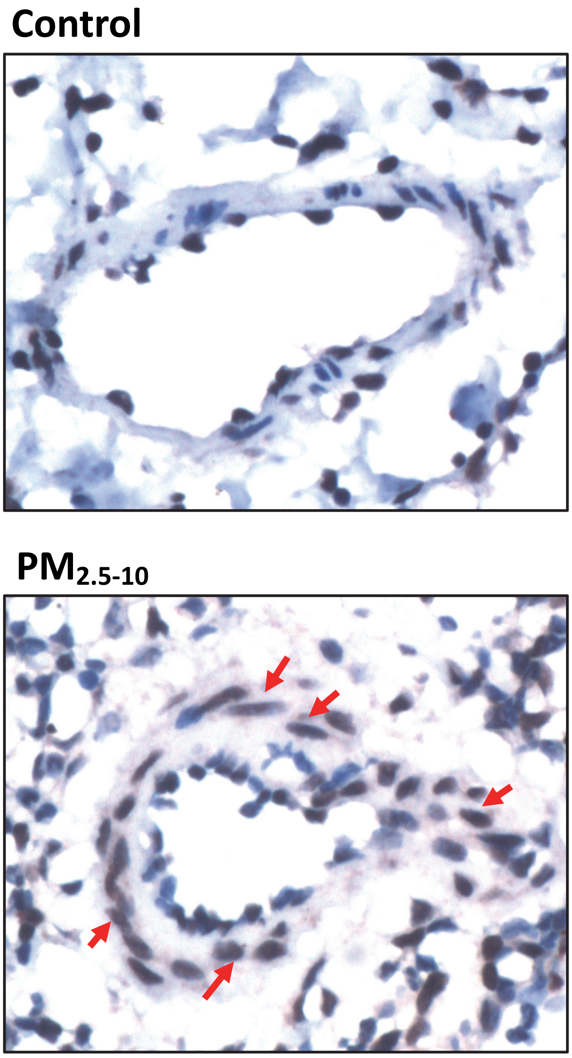

Figure S5. Immunohistochemical staining for NF-κB subunit phospho-p65 in small arteries of mouse lung. Mice were aspirated with 25 μg $PM_{2.5\text{-}10}$ per mouse, and twice per week for 2 weeks. Arrow indicates the phospho-p65 protein in the nuclei of MVSMCs in lung tissues. (brown color). The antibody was for phospho-p65 (GTX54672; GeneTex, Hsinchu, Taiwan).

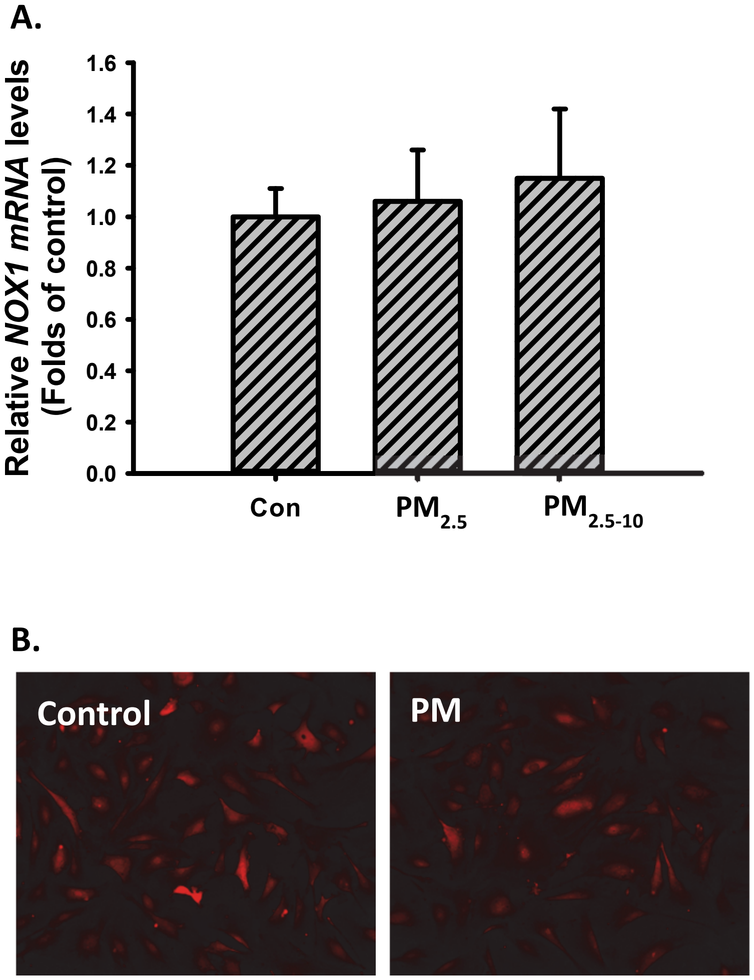

Figure S6. Effects of PM on the NOX1 mRNA and protein levels in MVSMCs.

MVSMCs were treated with d_2H_2O or 25 μg/mL PM for 24 h. (A) relative NOX-1 mRNA levels were determined. (B) Immunofluorescence staining for NOX-1 (GTX103888; GeneTex, Hsinchu, Taiwan)
